# 

**Published:** 1881-06

**Authors:** 


### CONTENTS FOR JUNE, 1881.

ORIGINAL COMMUNICATIONS.

ART. I.—On the Importance of the Early Recognition and the Repression of Mental Disease in its Incipient Stages. By EDWARD C. MANN, M. D. .... 299
ART. II.—Apocynum Cannabium in Bright's Disease. By T. S. DABNEY, M. D. .... 306
ART. III.—Beloved Physicians. By W. R. MONROE, M. D. .... 310

EDITORIAL DEPARTMENT.

At Our New Home .... 313
Special Notice .... 313
Craniotomy and its Alternatives .... 314
Code of Ethics .... 315
Hygiene .... 315
The Kidd-Quain Muddle .... 316
Treatment of Yellow Fever .... 316
Grindelia Robusta in Asthma .... 316
Quebracho .... 317
Salicine .... 317
Trichinosis .... 317
Quack Advertisements and the Religious Press .... 318
University of Maryland .... 318
American Medical Association .... 319
Medical Journal Association .... 319
Code of Ethics .... 320
The Business Management of the Independent Practitioner and the N. Y. Universal Purchasing Co. .... 321

BIBLIOGRAPHICAL DEPARTMENT.

A System of Oral Surgery .... 322
THERAPEUTIC PEARLS AT RANDOM STRUNG .... 323-325
SELECTIONS .... 325-347

POPULAR SCIENCE DEPARTMENT.

Anatomical and Physiological Differences between the Caucasian and African Races .... 348
Prof. Frank Donaldson, M. D., on Air and Ventilation .... 352
Opening of the Electric Railway in Berlin .... 356
What Air shall we Breathe at Night? .... 357
Cold Air for Domestic Uses .... 358
Antiquities in New Mexico .... 359
The Insulation of Electric Light Wires .... 360
Glacial Periods .... 361
Microscopic Structure of Metal .... 361
Grafting the Tomato on the Potato .... 362
Cooking by Electricity .... 363
How we are Poisoned .... 364
Anti-Septics and Disinfectants .... 367
The Lunar Display in Colorado .... 368
Treatment of Meniere's Disease .... 370
Cure of Goiter by Fluoric Acid .... 370
Radical Cure of Rupture .... 370
The Sunflower .... 370

